# Salvianolic Acid C against Acetaminophen-Induced Acute Liver Injury by Attenuating Inflammation, Oxidative Stress, and Apoptosis through Inhibition of the Keap1/Nrf2/HO-1 Signaling

**DOI:** 10.1155/2019/9056845

**Published:** 2019-05-12

**Authors:** Chien-Ta Wu, Jeng-Shyan Deng, Wen-Chin Huang, Po-Chou Shieh, Mei-Ing Chung, Guan-Jhong Huang

**Affiliations:** ^1^Faculty of Pharmacy, College of Pharmacy, Kaohsiung Medical University, Kaohsiung, Taiwan; ^2^Department of Health and Nutrition Biotechnology, Asia University, Taichung, Taiwan; ^3^Graduate Institute of Biomedical Sciences, School of Medicine, China Medical University, Taichung, Taiwan; ^4^Department of Chinese Pharmaceutical Sciences and Chinese Medicine Resources, College of Chinese Medicine, China Medical University, Taichung, Taiwan

## Abstract

Acetaminophen (APAP) overdose is one of the most common causes of drug-induced acute liver failure in humans. To investigate the hepatoprotective effect of salvianolic acid C (SAC) on APAP-induced hepatic damage, SAC was administered by daily intraperitoneal (i.p.) injection for 6 days before the APAP administration in mice. SAC prevented the elevation of serum biochemical parameters and lipid profile including aspartate aminotransferase (AST), alanine aminotransferase (ALT), total bilirubin (T-Bil), total cholesterol (TC), and triacylglycerol (TG) against acute liver failure. Additionally, SAC reduced the content of malondialdehyde (MDA), the cytochrome P450 2E1 (CYP2E1), and the histopathological alterations and inhibited the production of proinflammatory cytokines in APAP-induced hepatotoxicity. Importantly, SAC effectively diminished APAP-induced liver injury by inhibiting nuclear factor-kappa B (NF-*κ*B), toll-like receptor 4 (TLR4), and mitogen-activated protein kinases (MAPKs) activation signaling pathway. Moreover, SAC enhanced the levels of hepatic activities of glutathione (GSH), superoxide dismutase (SOD), glutathione peroxidase (GPx), catalase, and Kelch-like ECH-associated protein 1 (Keap1)/erythroid 2–related factor 2 (Nrf2)/heme oxygenase-1 (HO-1) pathway in APAP-induced mice. SAC mainly inhibited the activation of apoptotic pathways by reduction of cytochrome c, Bax, and caspase-3 protein expression. Taken together, we provide the molecular evidence that SAC protected the hepatocytes from APAP-induced damage by mitigating mitochondrial oxidative stress, inflammatory response, and caspase-mediated antiapoptotic effect through inhibition of the Keap1/Nrf2/HO-1 signaling axis.

## 1. Introduction

The liver plays a key role in the body's metabolism, detoxification, and secretion. Liver disease/failure is a very critical clinical issue among humans. It is well known that viruses, drugs, alcohol, toxic chemicals, and nutritional supplements can cause liver injury through direct or indirect toxicity related to the metabolites [1, 2]. Acetaminophen (N-acetyl-p-aminophenol (APAP)) is the most popular drug for the treatment of pain and fever that considered safe at the recommended therapeutic concentrations [3]. However, it is the risk of liver damage from taking too much or overdosed acetaminophen, leading to acute liver failure and even death.

APAP is one of the most commonly used analgesic and antipyretic drugs in the world [4]. APAP is relatively safe at therapeutic doses, but it is easily overused due to individual differences. In addition, liver damage caused by APAP overdose occurs rapidly within 24-48 hours after ingestion [5]. Currently, the clinical treatment of APAP-induced hepatotoxicity is extremely limited. N-acetylcysteine (NAC) was shown to be an effective first-line antidote for APAP poisoning and prevention of renal dysfunction, but its effectiveness is restricted to the early stages [6]. APAP is metabolized by glucuronidation or sulfation by cytochrome P450 2E1 (CYP2E1) into the reactive metabolite N-acetyl-p-benzoquinone imine (NAPQI). NAPQI depletes the cellular glutathione (GSH), resulting in severe oxidative stress and hepatocellular necrosis. However, when high dose of APAP is taken, a large amount of NAPQI is formed that causes the activation of the liver's innate immune cells and subsequent downstream of inflammatory mediators and proinflammatory cytokines to worsen the injury. Hepatocellular necrosis deteriorates with a decrease in the activity of antioxidative enzymes such as catalase, superoxide dismutase (SOD), or glutathione peroxidase (GPx) [7]. The nuclear factor erythroid 2–related factor 2 (Nrf2) is a major regulator of the antioxidant defense system that mediates cell survival and regulates the gene expression encoding intracellular detoxifying enzymes and antioxidant proteins via antioxidant response element (ARE) [7]. Nrf2-dependent ARE-driven genes encoding detoxification and antioxidant enzymes include NAD(P)H:quinone oxidoreductase 1 (NQO1), glutamate-cysteine ligase catalytic subunit (GCLC) and a modifier subunit (GCLM), and heme oxygenase-1 (HO-1) [8]. Therefore, this reaction process increases reactive oxygen species (ROS) and lipid peroxidation, ultimately leading to hepatic apoptosis.

Liver damage triggers Kupffer cell activation (the phagocytic macrophages of the liver) that led to increase oxidative stress and inflammatory mediators (TNF-*α*, IL-1*β*, and IL-6) and activate other inflammatory cells (infiltrating macrophages and neutrophils) [8]. Currently, the progression of APAP-induced liver damage remains unclear. There is increasing evidence that the mediators of oxidative stress and inflammation are associated with the toxifying process of APAP-induced liver damage.

The roots of *Salvia miltiorrhiza* Bunge (Labiatae) have traditionally been applied in clinic used to treat inflammatory diseases and cardiovascular diseases. It contains two major classes of biologically active compounds, tanshinones and salvianolic acids. Tanshinones are a lipophilic pigment that found to have potent anticancer, antiatherosclerotic, and antihypertensive activities [9]. Salvianolic acids are water-soluble active components, which are mainly taking responsibility for the beneficial activities of inflammatory and cardiovascular diseases [10]. Salvianolic acid C (SAC) is an organic compound that consists of two units of tashinol and a single unit of caffeic acid. SAC can reduce hypolipidemic activity by inhibiting human HMG-CoA reductase activity [11]. In addition, SAC significantly induced apoptosis in hepatoma cancer cells [12] and inhibited NF-*κ*B activity in endothelial cells [13]. In the present study, we evaluated the protective effects of SAC on APAP-induced hepatotoxicity and determined the molecular mechanism by which SAC inhibited oxidative stress, inflammation, and caspase-mediated antiapoptotic effect in a mouse model.

## 2. Material and Methods

### 2.1. Chemicals

**Figure 1:**
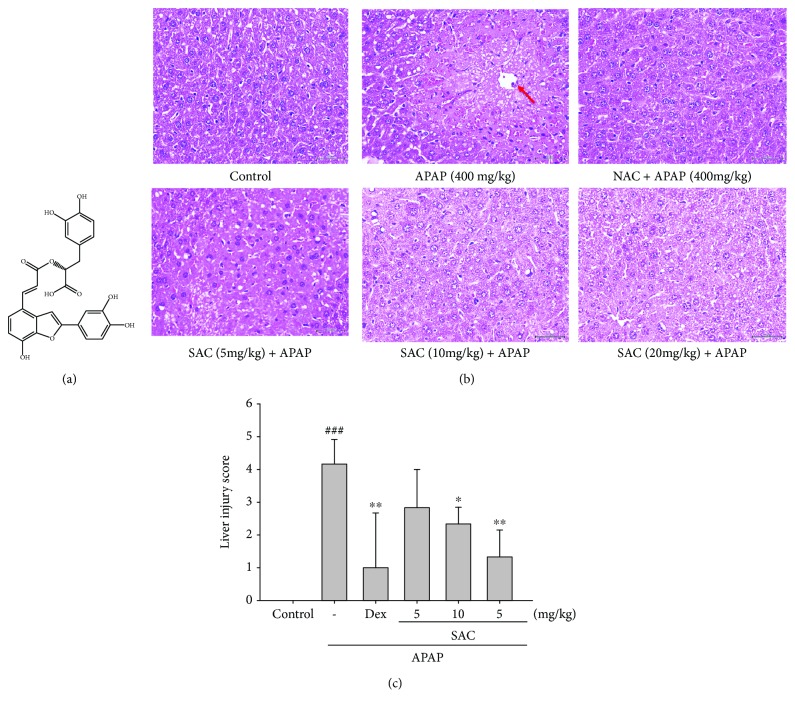
Structure of salvianolic acid C (SAC) (a) and the effects of SAC on histopathological changes in the liver (b) and on the severity of liver injury were analyzed using the liver injury scoring system (c) in APAP-induced mice. Sections were stained with H&E (400x) and observed under a light microscope. The liver was excised and embedded in 10% formalin, sectioned, and stained with H&E; magnification ×400. The images are the representative of three experiments. NAC: N-acetyl cysteine. The data are presented as the means ± S.E.M. ^##^ compared with the control group. ^∗^ *p* < 0.05 and ^∗∗^ *p* < 0.01 compared with the LPS-alone group. Arrowhead donated central veins and highlight liver injury/necrosis.

APAP, N-acetylcysteine (NAC), and other chemicals were delivered by Sigma-Aldrich Chemical Co. (Steinheim, Germany). Salvianolic acid C (Figure 1(a); purify 98.6%) was purchased from Chem Faces Pharmaceutical Company (Wuhan, China). The amounts of mouse TNF-*α*, IL-1*β*, and IL-6 ELISA Max™ Set Deluxe Kits were received from BioLegend Inc. (San Diego, CA, US). Primary antibodies against COX-2, p-JNK, catalase, GPx, SOD, Keap1, NQO1, GCLC, and GCLM were purchased from GeneTex (San Antonio, TX, USA). Antibodies against PI3k/p-AKT were purchased from Merck Millipore (Sigma-Aldrich; Merck KGaA, Darmstadt, Germany). Antibodies against TLR4, iNOS, NF-*κ*B, I*κ*B*α*, p38, HO-1, Nrf-2, and *β*-actin were purchased from Abcam (Cambridge, UK, USA). Antibodies against JNK, p-ERK, ERK, p-p38, and p-I*κ*B-*α* were purchased from Cell Signaling Technology (Beverly, MA, USA). Protein assay kit (Bio-Rad Laboratories Ltd., Watford, Herts, UK) was obtained as indicated. Poly-(vinylidene fluoride) (PVDF) membrane (Immobilon-P) was obtained from Millipore Corp. (Bedford, MA, USA).

### 2.2. Animals

Adult male ICR mice (6–8 weeks old) were used from BioLASCO Taiwan Co., Ltd. (Taipei, Taiwan). All animal experiments were carried out in accordance with the regulations of the Animal Management Committee of China Medical University (IACUC approval number: 2018-286). Every effort is made to minimize the number and suffering of animals used in these experiments.

### 2.3. Experimental Protocol

After 1-week adaptive breeding, mice were randomly divided into the following six groups (*n* = 6): control, APAP (400 mg/kg), APAP + NAC (600 mg/kg), and APAP + SAC (5 mg/kg, 10 mg/kg, and 20 mg/kg). SAC was injected into the mice in the three experimental groups for 6 consecutive days. Normal and APAP mice were treated with PBS in the same manner. One hour after the final SAC dose treatment, mice were administered with APAP (400 mg/kg) by a single intraperitoneal injection in all groups (except the control group). Mice were euthanized at 12 h post-APAP challenge, and blood samples were collected [14].

### 2.4. Measurement of Hepatic Injury

Blood was centrifuged (5 min at 12000 g) to separate the serum. The serum levels of ALT, AST, T-Bil, TC, and TG were determined by following the instructions on the commercial kits (HUMAN Diagnostics Worldwide, Germany).

### 2.5. Histology

The liver tissue fixation method that was in 10% formalin for 24 h were processed at room temperature before paraffin embedding. The sections were stained with hematoxylin and eosin (H&E) for morphological evaluation. The sections were examined using a Nikon compound microscope (Nikon, ECLIPSE, TS100, Japan). The severity of liver injury scores from one to five depends on the degree of necrosis, coagulative, central area, and focal. Degree of lesions was graded from one to five depending on severity: a score of 0 expressed normality, 1 expressed minimal (<1%), 2 expressed slight (1-25%), 3 expressed moderate (26-50%), 4 expressed moderate/severe (51-75%), and 5 expressed severe/high (76-100%) [14].

### 2.6. Lipid Peroxidation Assay

MDA is estimated by the thiobarbituric acid reactive substance (TBARS). The extract of sample was combined with the thiobarbituric acid (TBA) reagent (two parts 0.4% TBA in 0.2 M HCl and 0.2% butylated hydroxytoluene (BHT) in 95% ethanol). The mixture was placed in a water bath at 90°C for 45 min and cooled, and n-butanol was added (1 : 1). After the centrifugation, carefully transfer the supernatant to the new tube. The solution was recorded at 535 nm [15].

### 2.7. Glutathione Estimation

GSH was estimated according to the modified method [16]. The liver tissues were homogenized with 10% TCA buffer and centrifuged at 3000 rpm for 10 min at 4°C. The reaction mixture contained 0.1 mL of supernatant, 2.0 mL of 0.3 M phosphate buffer (pH 8.4), 0.4 mL of double-distilled water, and 0.5 mL of DTNB (5,5-dithiobis (2-nitrobenzoic acid)). OD was read (within 2-3 min after the addition of DTNB) at 412 nm against a reagent blank. Absorbance values were compared with a standard curve generated from known GSH. The concentration of GSH was expressed as *μ*mol/g tissue.

### 2.8. Nitrites Assay

Determination of nitrite level in serum was indirectly assessed by the Griess reagent [17]. For a moment, add the equal volume of Griess reagent, and serum was mixed. After 10 min of incubation, the absorbance of supernatants was measured using a microplate spectrophotometer at 540 nm.

### 2.9. Measurement of Serum TNF-*α*, IL-1*β*, and IL-6 Levels

Inflammatory profiles (TNF-*α*, IL-1*β*, and IL-6) of serum were evaluated by enzyme-linked immunosorbent assay (ELISA) system according to the manufacturer's instructions. The concentrations were expressed as pg/mL. The optical density was measured at 450 nm on an automated ELISA reader (VersaMax, Molecular Devices, CA, USA).

### 2.10. Western Blotting Analysis

Liver tissue (30-50 mg) was prepared into homogenate samples and lysed in RIPA lysis buffer, followed by centrifugation (12000×g, 20 min) and the protein concentration determined by a Bio-Rad protein assay kit (Bio-Rad, Hercules, CA). Fifty micrograms of protein per lane were loaded on 10% SDS-PAGE gels and transferred onto polyvinylidene fluoride (PVDF) membranes (Millipore, Bedford, MA, USA). Membranes are further blocked and incubated overnight with primary antibodies at 4°C using 1 : 2000 dilutions. Then, appropriate horseradish peroxidase- (HRP-) conjugated secondary antibodies (Sigma, St. Louis, MO, US) were applied at room temperature, and signals were detected by enhanced chemiluminescence (ECL) reagent (Amersham International plc., Buckinghamshire, UK). Western blot analysis was analyzed using Kodak Molecular Imaging Software (Eastman Kodak Company, Rochester, NY) and shown in the relative intensities.

### 2.11. Statistical Analysis

All values were expressed as mean ± standard error of the mean (S.E.M.). One-way analysis of variance (ANOVA) or Student's *t*-test was used to examine the differences among multiple groups or between two groups. ^###^ denotes *p* < 0.001 compared with the control group; ^∗^ denotes *p* < 0.05, ^∗∗^ denotes *p* < 0.01, and ^∗∗∗^ denotes *p* < 0.001 significant compared to APAP-alone group.

## 3. Results

### 3.1. Liver Histopathology

According to the histopathological image analysis, APAP toxicity is the leading cause in the liver morphological changes, including steatosis, lobular inflammation, hepatocyte necrosis, and hepatocyte ballooning (Figure 1(b)). SAC definitely alleviated the liver failure with reduced hepatocyte necrosis and liver cell degeneration. Furthermore, the lung injury score showed that SAC improved the APAP-induced inflammatory response (Figure 1(c)). These results suggested that SAC protected the APAP challenge histopathological changes through the reduction of hepatocyte necrosis and liver cell degeneration of liver tissues in the mice.

### 3.2. Effect of SAC on Serum Biochemical Markers

**Figure 2:**
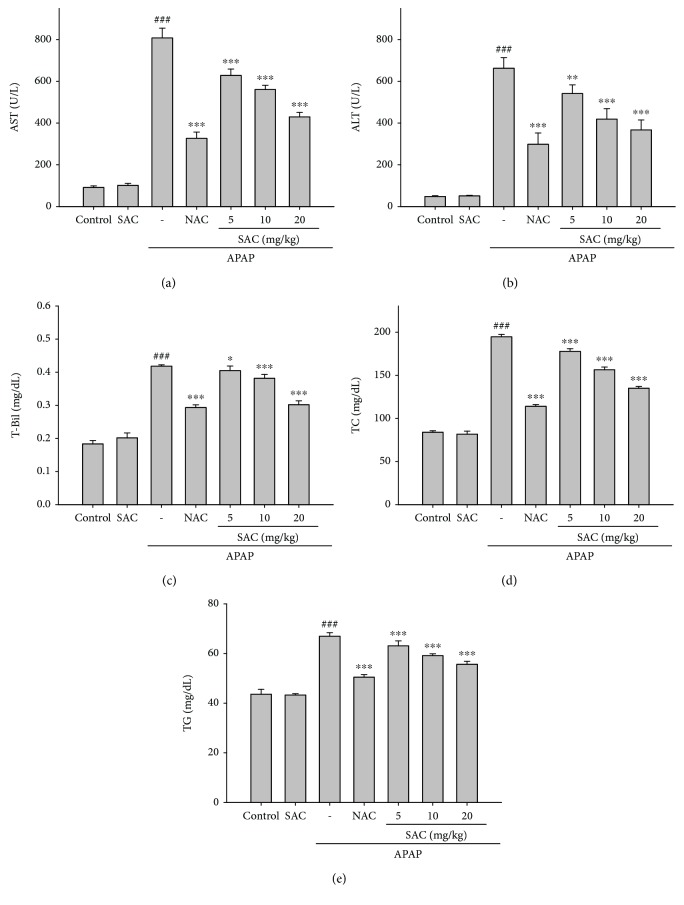
Effects of SAC on serum AST (a), ALT (b), T-Bil (c), TC (d), and TG (e) in APAP-induced mice. Mice were given PBS and SAC (20 mg/kg) alone or SAC (5, 10, and 20 mg/kg body weight) via intraperitoneal injection 1 h before challenge with APAP (400 mg/kg). Mice were killed at 12 h after APAP challenge. The values are reported as the means ± S.E.M. of six mice per group. ^###^ *p* < 0.01 compared with the control group; ^∗^ *p* < 0.05, ^∗∗^ *p* < 0.01, and ^∗∗∗^ *p* < 0.001 compared with the APAP group.

To assess the mouse liver damage affected by APAP, SAC, and NAC, several serum biochemical markers (AST, ALT, and T-Bil) of liver failure were measured. Increased levels of AST, ALT, and T-Bil in all animals treated with APAP compared to the control group were observed (Figures 2(a)-2(c)). SAC (5, 10, and 20 mg/kg) and NAC (600 mg/kg) significantly avoided the increase in the serum AST, ALT, and T-Bil levels. Moreover, the APAP-induced mice had elevated the levels of serum TC and TG (Figures 2(d) and 2(e)). The serum TG and cholesterol levels were reduced after the SAC treatment, significantly. Collectively, these results demonstrated that SAC plays an important role in the regulation of the hepatoprotective activity compared to the APAP-induced liver injury group. In addition, we measure the effect of SAC alone (20 mg/kg) without the treatment of APAP. The results have not affected the level of biochemical parameters including AST, ALT, T-Bil, TC, and TG.

### 3.3. Inhibition of APAP-Induced Lipid Peroxidation by SAC

**Figure 3:**
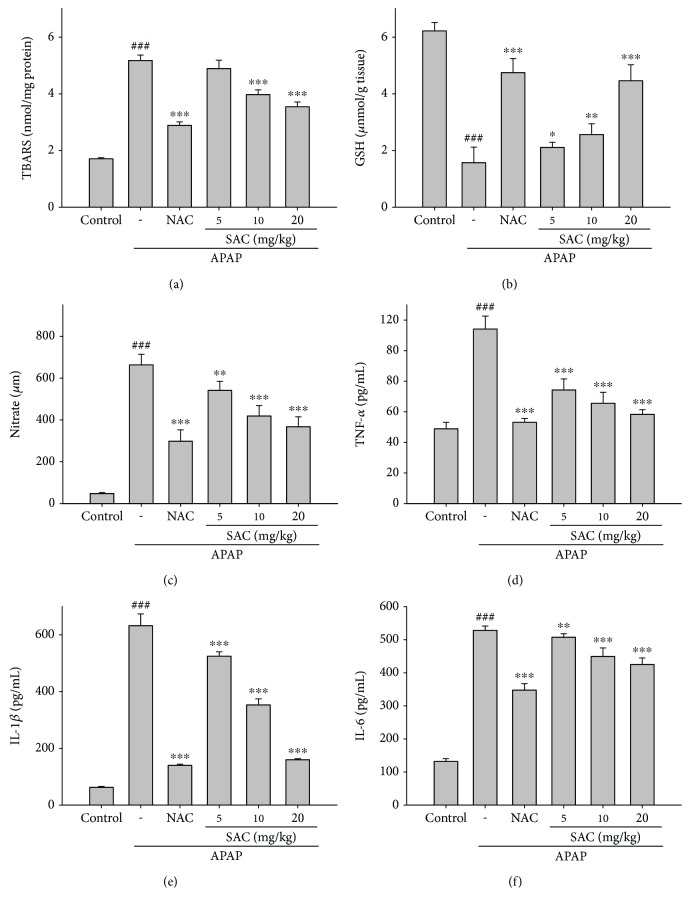
Effects of SAC on liver lipid peroxides (a) and GSH (b) levels and on serum NO (c), TNF-*α* (d), IL-1*β* (e), and IL-6 (f) levels in APAP-induced mice. Mice were given PBS alone or SAC (5, 10, and 20 mg/kg body weight) via intraperitoneal injection 1 h before challenge with APAP (400 mg/kg). Mice were killed at 12 h after APAP challenge, and the liver and serum were harvested. GSH was determined and expressed as *μ*mol/g liver tissues. Nitrite concentration in the serum was determined using Griess reagent. Serum concentrations of TNF-*α*, IL-1*β*, and IL-6 were determined by the commercial ELISA kits. The values are reported as the means ± S.E.M. of five mice per group. ^###^ *p* < 0.01 compared with the control group; ^∗^ *p* < 0.05, ^∗∗^ *p* < 0.01, and ^∗∗∗^ *p* < 0.001 compared with the APAP group.

Assessment of lipid peroxidation in hepatic tissues was used to determine the TBARS levels, which are the indicators of lipid peroxidation. APAP-induced hepatotoxicity examined a significant increase in the levels of TBARS (Figure 3(a)). SAC diminished the levels of TBARS in the APAP-intoxicated group at different concentrations. These results indicate that the hepatoprotective effect of SAC might also be due to the inhibition of lipid peroxidation in the liver.

### 3.4. Effect of SAC on the Levels of GSH

Previous studies showed that oxidative stress was closely associated with liver injury [16]. As shown in Figure 3(b), APAP administration significantly reduced the levels of GSH in hepatic tissues. However, when compared with the model group, pretreatment with SAC and NAC markedly reversed these trends. In addition, we measure the effect of SAC alone (20 mg/kg) without the treatment of APAP. The results have not affected the level of GSH (Figure 2S).

### 3.5. Inhibition of APAP-Induced Liver Inflammation

**Figure 4:**
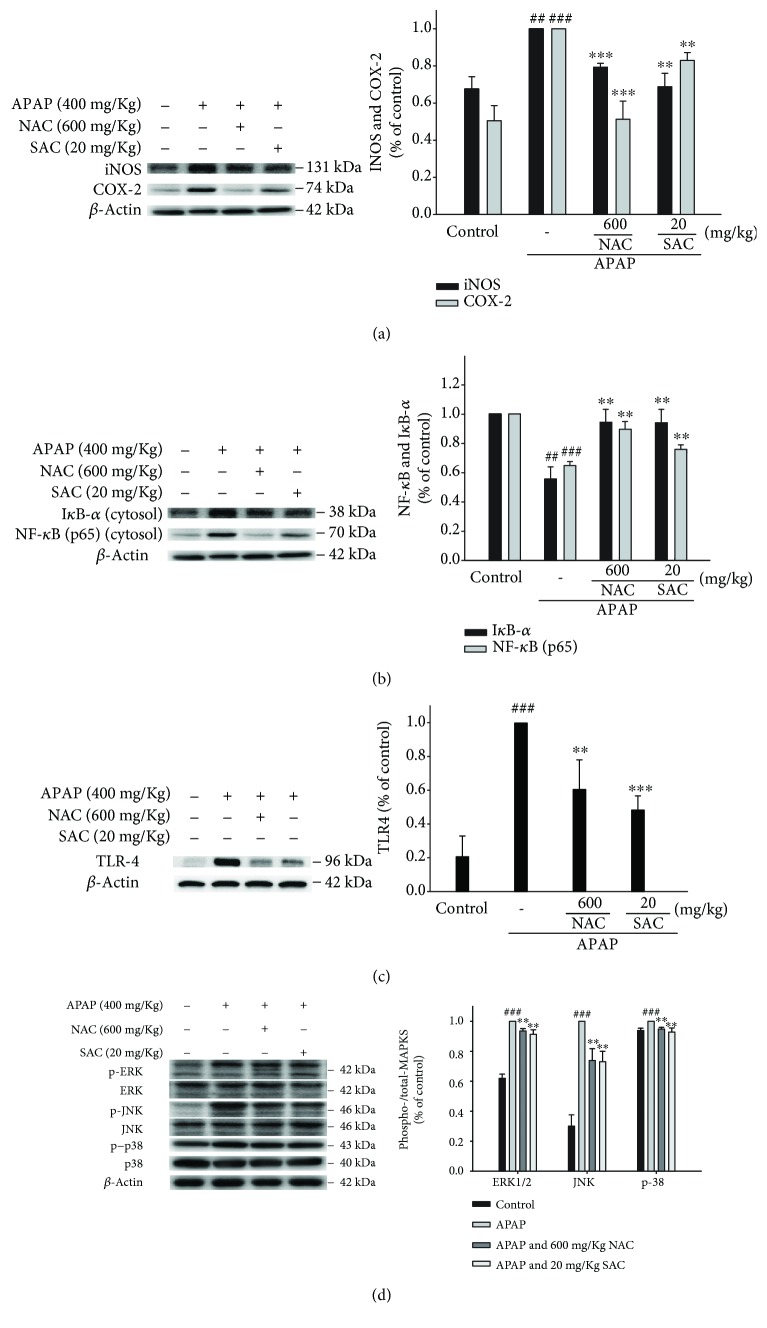
SAC inhibited iNOS, COX-2 (a), I*κ*B*α*, NF-*κ*B (b), TLR4 (c), and MAPK (d) expression in APAP-induced mice. Total protein was extracted from liver tissues, and the protein levels of iNOS, COX-2, I*κ*B-*α*, NF-*κ*B, TLR4, p-JNK, JNK, p-ERK, ERK, p-p38, and p-38 were determined by Western blot analysis. *β*-Actin served as a loading control. The values are reported as the means ± S.E.M. of five mice per group. ^###^ *p* < 0.01 compared with the control group; ^∗^ *p* < 0.05, ^∗∗^ *p* < 0.01, and ^∗∗∗^ *p* < 0.001 compared with the APAP group.

Inflammation is the source of acute liver damage. As depicted in Figures 3(b)-3(d) and 4(e), the levels of serum NO, TNF-*α*, IL-1*β*, and IL-6 exhibited a significantly higher injury in APAP-induced hepatotoxicity. SAC also suppressed the release of these cytokines, which may partially diminish the inflammatory injury in the liver, significantly. These results indicate that the hepatoprotective effect of SAC might also be due to its capacity to inhibit the potent anti-inflammatory cytokines.

### 3.6. Inhibition of APAP-Induced Hepatotoxicity iNOS and COX-2 and Inactivation of NF-*κ*B and I*κ*B*α* Protein Expression

As depicted in Figure 4(a), the protein expression of iNOS and COX-2 was increased compared to the control group after APAP challenge. SAC and NAC treatment significantly reduced the protein expression of iNOS and COX-2 in APAP-induced acute hepatic injury.

The NF-*κ*B pathway has been demonstrated to regulate the production of various proinflammatory cytokines in the nucleus, including TNF-*α*, IL-1*β*, and IL-6. We next assessed the expression of NF-*κ*B and I*κ*B*α* in APAP-induced liver damage by using a Western blot. As shown in Figure 4(b), SAC treatment suppressed both NF-*κ*B and I*κ*B*α* degradation in the cytosol after APAP challenge. It suggests that SAC mediated the NF-*κ*B pathway in the APAP-induced liver damage model.

### 3.7. Effect of SAC on APAP-Induced Activation of TLR4 and MAPKs

Many lines of evidence indicate increased expression of TLR4 in the liver of APAP-toxic mice [14]. APAP administration significantly induced the TLR4 expression levels compared to the untreated control group, whereas SAC pretreatment significantly inhibited these elevations (Figure 4(c)), implying the downregulated NF-*κ*B-mediated inflammatory cascade. In addition, as shown in Figure 4(d), SAC treatment effectively suppressed the phosphorylation of ERK, JNK, and p38 proteins after APAP challenge. According to the previous reports [14], the inflammatory cytokines downregulated the MAPK and NF-*κ*B signaling pathway. SAC also can inhibit the NF-*κ*B pathway and inflammatory response by inhibiting the activation of MAPKs. Thus, these in vivo results suggest that SAC may protect APAP-induced hepatotoxicity by inactivation of the TLR4/MAPK/NF-*κ*B signaling pathway.

### 3.8. Attenuation of APAP-Induced Oxidative Stress and the Keap-1/Nrf2/HO-1 Signal Pathway by SAC

**Figure 5:**
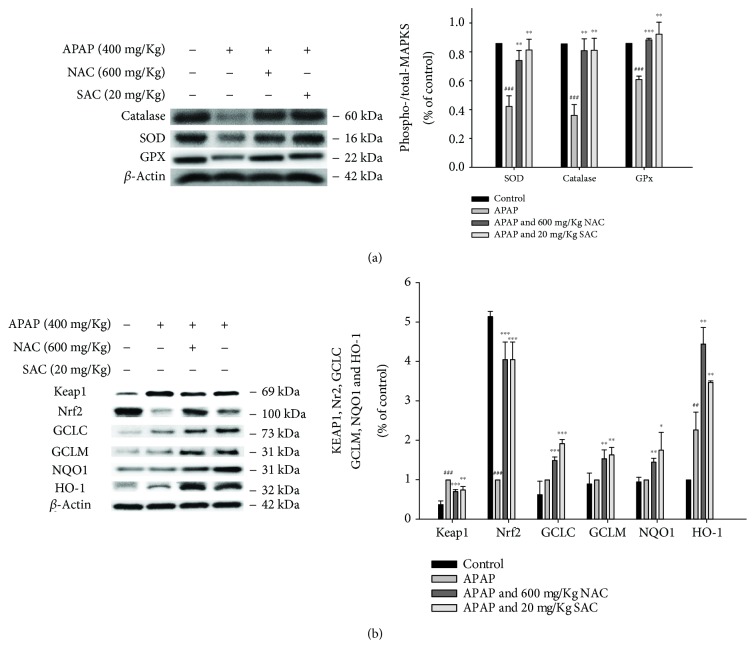
SAC inhibited antioxidant enzymes (catalase, SOD, and GPx) (a) and detoxification capacities via the Nrf2/Keap1 signaling pathway (b) in APAP-induced acute liver injury in mice. Total protein was extracted from liver tissues, and the protein levels of catalase, SOD, GPx, HO-1, Nrf2, Keap1, NQO1, GCLC, and GCLM were determined by Western blot analysis. *β*-Actin served as a loading control. The values are reported as the means ± S.E.M. of five mice per group. ^###^ *p* < 0.01 compared with the control group; ^∗^ *p* < 0.05, ^∗∗^ *p* < 0.01, and ^∗∗∗^ *p* < 0.001 compared with the APAP group.

Oxidative stress causes liver tissue damage by the excess ROS production and inhibits the activity of hepatic antioxidant defense enzymes, including SOD, catalase, and GPx. We observed that the protein expression of these antioxidant enzymes was decreased in APAP-induced hepatotoxicity compared to the control. Additionally, SAC and NAC increased the expression of SOD, catalase, and GPx compared with the APAP-treated mice (Figure 5(a)). These data indicate that SAC enhanced the antioxidant defense system to prevent APAP-induced oxidative damage.

The molecular mechanism of activation of the Keap1/Nrf2/HO-1 signaling pathway provides a new therapeutic strategy for the improvement of various inflammatory diseases. To investigate whether the Nrf2 signaling pathway is involved in the hepatoprotective effect of SAC, the expression of Nrf2 and signaling pathway-related proteins was determined by Western blotting. As shown in Figure 5(b), SAC can result in a decrease in cytoplasmic levels in APAP-induced acute liver injury. In addition, the protein expression of Keap1 was significantly increased by APAP and was reduced by SAC.

APAP administration resulted in a decrease in Nrf2 and its target downstream proteins such as GCLC, GCLM, NQO1, and HO-1, and these changes were completely reversed by SAC (Figure 5(b)). The above results indicate that SAC can regulate the expression of detoxification enzymes and antioxidant proteins that play a hepatoprotective effect. SAC might to some extent promote Nrf2 nuclear translocation and its target gene expression. These data indicate that SAC increases the Nrf2 protein accumulation, leading to an enhancement of the antioxidant defense system to prevent APAP-induced oxidative damage.

### 3.9. Suppression of CYP2E1 Expression by SAC

**Figure 6:**
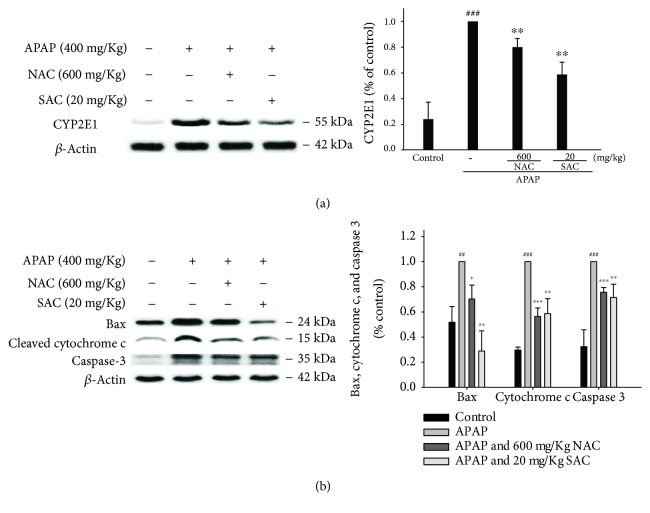
SAC inhibited CYP2E1 (a), cleaved caspase-3, Bax, and cytochrome c (b) protein expression in APAP-induced mice. Total protein was extracted from liver tissues, and the protein levels of CYP2E1, cleaved caspase-3, Bax, and cytochrome c were determined by Western blot analysis. *β*-Actin served as a loading control. The values are reported as the means ± S.E.M. of five mice per group. ^###^ *p* < 0.01 compared with the control group; ^∗^ *p* < 0.05, ^∗∗^ *p* < 0.01, and ^∗∗∗^ *p* < 0.001 compared with the APAP group.

CYP2E1 is a key enzyme that metabolizes xenobiotics and can trigger a series of events that lead to APAP-induced liver toxicity. Therefore, inhibition of CYP2E1 expression may lead to a reduction of liver tissue damage. As shown in Figure 6(a), SAC decreased the expression of CYP2E1 in the APAP-treated group. A positive result indicates protective effects of SAC against APAP-induced hepatotoxicity through inhibition of CYP2E1 in mice. In addition, we also measure the effect of SAC alone (20 mg/kg) without the treatment of APAP. The results have not affected the expression of CYP2E1 (Figure 3S).

### 3.10. Effect of SAC on the Expressions of Bax, Cleaved Caspase-3, and Cytochrome c in APAP-Treated Mice Liver

The main cause of massive mitochondrial dysfunction and hepatocyte apoptosis is APAP overdose. As depicted in Figure 6(b), APAP significantly induced Bax mitochondrial translocation, released cytochrome c, and activated cleavage caspase-3. In addition, SAC pretreatment suppressed the expression of Bax, cytochrome c, and cleavage caspase-3 after compared to the APAP-treated group. Taken together, SAC pretreatment prevented the APAP-induced DNA damage.

## 4. Discussion

An acute overdose of acetaminophen-induced fatal hepatotoxicity is characterized by exceedingly the accumulation of multiple indices of cellular damage from oxidative stress and ER stress [17]. This triggers an inflammatory response of APAP by activating Kupffer cells that contribute to in by producing proinflammatory cytokines and mediators in APAP-induced liver injury [18]. Two crucial factors of oxidative stress accumulation and inflammatory responses result in massive hepatocyte necrosis and liver failure in APAP-induced liver injury [19]. NAC approved antidote for an APAP overdose in the clinical patients, but it is only effective during the early period [20]. Therefore, it is still necessary for patients with overdosed APAP to find some effective undeveloped drugs and explore their potential molecular mechanism.

The results of this study showed that preoral SAC-treated APAP-treated mice significantly altered histology, including necrotic hepatocytes around the central vein, infiltrated with inflammatory cells, degeneration of hepatocytes, and proliferation of mononuclear phagocytes. In addition, SAC displayed the same effect as a positive control of NAC. SAC prevented the elevation of AST, ALT, and T-Bil and inhibited abnormal lipid metabolism (TC and TG) in the serum caused by impaired liver function after an overdose of APAP. ALT and AST are enzymes made by liver cells and will be released into the circulation when liver cells are damaged [21]. In addition, administration of APAP increases the levels of lipid peroxidation products which have been demonstrated to be associated with oxidative stress. Excessive oxidative stress triggers lipid peroxidation and results in the destruction of cellular components and cell death [22]. These data suggest that SAC can effectively protect APAP-induced liver injury as well as improve biochemical parameters and reduce the lipid oxidation.

NAPQI-induced GSH depletion plays a key role in APAP toxicity and also inhibits antioxidant enzyme activity to increase oxidative stress [23]. Our results showed that APAP significantly reduces GSH content, increases TBARS levels, and also inhibits antioxidant enzyme activity (SOD, catalase, and GPx), suggesting that APAP induces redox imbalance to accumulate ROS, lipid peroxidation, and formation of protein oxidation products. However, after pretreatment with SAC, the levels of GSH and TBARS were normalized and antioxidant enzyme activity was restored in the liver. These results suggested that SAC pretreatment protects against APAP-induced hepatotoxicity by reducing ROS.

After exposure to various hepatic toxins, the liver plays an important role in the process of detoxification. Inflammatory reactions expand tissue damage and lead to incorrect tissue repair, and it is initiated by the release of proinflammatory cytokines [24]. Our study showed that APAP-induced hepatotoxicity increased the levels of proinflammatory cytokines, and SAC treatment significantly reduced the expression of proinflammatory mediators of APAP-induced liver inflammation.

Proinflammatory cytokines are produced by activated immune cells, which increases the protein expression of iNOS and COX-2 and the activation of NF-*κ*B after APAP challenge to further aggravate liver injury [25]. Overproduction of NO via the iNOS has a cytotoxic effect and plays a role in many physiological processes [25]. Thus, the expression of iNOS acts as a regulator of APAP-induced hepatotoxicity. Induction of COX-2 is also found in an experimental model of APAP-induced hepatotoxicity, namely, NO-induced decrease and the expression levels of iNOS and COX-2 proteins which were inhibited that linked with the oxidative stress-induced inflammation-related chronic diseases. In addition, NF-*κ*B induces the various proinflammatory expressions that play a major role in the pathological changes in the liver [26]. Activation of NF-*κ*B protein is associated with APAP attack, which promotes the expression of TNF-*α* and COX-2 [27]. Therefore, SAC can prevent APAP-induced liver injury through the NF-*κ*B-mediated inflammation response.

TLR acts as an important regulator of exogenous pathogen-associated molecular patterns (PAMP) and damage-associated molecular patterns (DAMP) that recognize stress or dying cell release during tissue injury [28]. Several reports have revealed the involvement of activated TLR4 in APAP-intoxicated mouse livers [7, 8]. Our data show that APAP administration results in increased expression of TLR4, which in turn promotes activation of MAPK and NF-*κ*B and subsequent production of inflammatory mediators and proinflammatory factors, ultimately leading to exacerbated liver damage. However, SAC pretreatment significantly restored all changes, indicating that SAC may have a hepatoprotective effect on APAP-induced acute liver injury.

The ERK, JNK, and P38 MAPK family play an important role in the production of ROS and proinflammatory cytokines [28]. Overall, activation of JNK leads to mitochondrial dysfunction, which leads to hepatocyte apoptosis in APAP-induced liver injury [29]. Studies have also found that cells can regulate hepatotoxicity by regulating proinflammatory cytokines and anti-inflammatory cytokines via the ERK pathway after APAP challenge [30]. In addition, inflammatory mediator production was associated with the activation of the p38-MAPK pathway. Due to this combination, SAC effectively prevented hepatic injuries after APAP challenge by the phosphorylated MAPK levels.

Oxidative stress is highly correlated with APAP-induced hepatotoxicity that promotes ROS and NO overproduction because APAP-induced acute liver failure reduced antioxidant enzyme expression [31]. SOD, CAT, and GPx are antioxidant enzymes protecting cells from oxidative stress [32]. The Nrf2/Keap1 pathway is a key cellular defense mechanism that plays a key role in the regulation of phase II detoxifying and antioxidant enzymes in preventing APAP-induced acute liver failure [33]. Thus, the mechanism of APAP-induced hepatotoxicity is a synergistic effect on the accumulation of ROS and decrease of Nrf2-mediated defense responses [28]. In addition, Nrf2 can be transferred to the nucleus and be bound to ARE, regulating the expression of downstream genes involved in antioxidant and detoxification [31]. GCLC, GCLM, NQO1, and HO-1 are phase II enzymes regulated by Nrf2. GCL, composed of the catalytic subunit GCLC and the modified subunit GCLM, is a rate-limiting enzyme that regulates GSH biosynthesis [32]. The induction of these two proteins by SAC is accompanied by an increase in GSH levels, which may explain how SAC/APAP cotreatment can increase GSH levels. These observations provide evidence that SAC upregulates GCLC and GCLM via the Nrf2 signaling pathway leading to an increase of GSH synthesis. NQO1 has the ability to reduce NAPQI and improve APAP toxicity [31]. Moreover, Nrf2 is an inhibitor regulated by a cytosolic Keap1 protein, and the decreased Nrf2/Keap1 protein interaction led to increased Nrf2 expression. Nrf2/Keap1 plays important roles in APAP-induced hepatotoxicity. HO-1 was induced by a wide range of stress conditions such as oxidative stress and inflammatory signals. Thus, the potential role of induction of HO-1 was in cellular antioxidant defense, and it is regulated by Nrf2. Nrf2 also has a crucial role in maintaining cellular redox homeostasis that allows adaptation and survival against mitochondrial damage and cell death. HO-1/Nrf2 signals have potentials to avoid the drug-induced hepatocellular injury [32]. In this study, we showed that SAC stimulated the expression of HO-1 and Nrf2 proteins by reducing oxidative damage and regulating antioxidative enzymes to protect against APAP-induced hepatotoxicity.

Overdosed APAP caused hepatotoxicity by inhibiting cytochrome P450 enzymes, particularly CYP2E1, to form NAPQI. Excess NAPQI depletes GSH and reacts with proteins to form complexes, resulting in impaired mitochondrial function and increased the generation of ROS and DNA damage. CYP2E1-deficient mice are resistant to hepatotoxicity induced by high dose of APAP [34, 35]. As a result, mitochondrial permeability transition inhibits ATP synthesis and triggers cell necrosis. Thus, CYP2E1 plays a role in controlling APAP-induced liver injury. Inhibition of CYP2E1 expression may reduce the production of NAPQI, which is the main target of APAP hepatotoxicity prevention or therapeutic intervention.

Apoptosis of hepatocytes plays an important role in the APAP hepatic damage model, and suppressing hepatic apoptosis would diminish the development of acute liver failure [36]. Several proteins are included of hepatocyte apoptosis such as caspase-3, Bax, and cytochrome c that inhibit mitochondrial DNA damage and cell death through mitochondrial antioxidant and redox states. Bax is a proapoptotic protein that translocates to the mitochondria upon the induction of apoptosis by inducing mitochondrial membrane permeabilization with the release of cytochrome c. Cytochrome c is released into the cytoplasm, which is considered a sign of severe mitochondrial damage [37]. In this study, we demonstrated that SAC protected the hepatocytes from apoptosis by blockade of the APAP-triggered mitochondrial apoptotic pathway via suppressing the expression of Bax and caspase-3 and inhibition of the release of cytochrome c.

## 5. Conclusions

**Figure 7:**
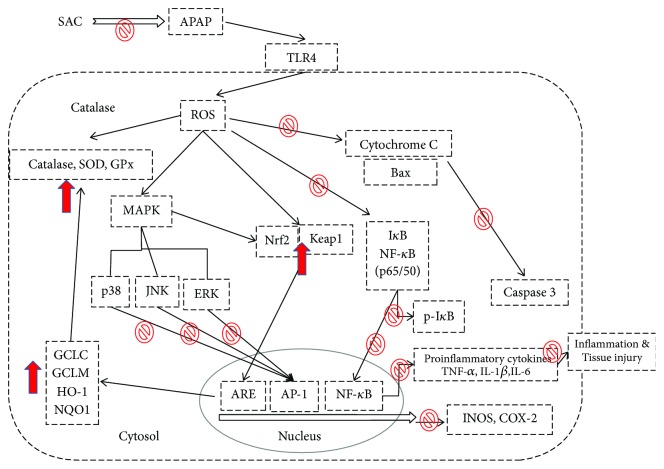
The schemes of the mechanism for the protective effect of SAC on APAP-induced liver injury.

The present study demonstrated that SAC displayed a significant therapeutic effect in alleviating hepatic toxicity in APAP-induced mice and can improve hepatic dysfunction and inhibit histopathological changes. Additionally, SAC has an effective hepatoprotective activity, and its mechanism would be able to contribute to the inhibition of oxidative stress, inflammation, and apoptosis (Figure 7). These results provide a rationale that SAC can be applied as a potential therapy to protect and treat liver disease and damage in the near future.

## Data Availability

The data used to support the findings of this study are available from the corresponding author upon request.

## Supplementary Materials

Supplementary Materials Figure 1S: effects of salvianolic acid C (SAC) on lipopolysaccharide- (LPS-) induced cell viability (A) and NO production (B) of RAW 264.7 macrophages. Cells were incubated for 24 h with 1 *μ*g/mL of LPS in the absence or presence of SAC (0, 2.5, 5, 10, and 20 *μ*g/mL). SAC was added 1 h before incubation with LPS. Cell viability assay was performed using MTT assay. Nitrite concentration in the medium was determined using Griess reagent. The data were presented as mean ± SD for the three different experiments performed in triplicate. ^###^ compared with a sample of the control group (one-way ANOVA followed by Scheffe's multiple range tests). ^∗^ *p* < 0.05 and ^∗∗∗^ *p* < 0.001 were compared with LPS-alone group. Figure 2S: effects of SAC on GSH level in APAP-induced mice. Mice were given PBS alone or SAC (5, 10, and 20 mg/kg body weight) via intraperitoneal injection 1 h before challenge with APAP (400 mg/kg). Mice were killed at 12 h after APAP challenge and the liver. GSH was determined and expressed as *μ*mol/g liver tissues. The values are reported as the means ± S.E.M. of five mice per group. ^###^ *p* < 0.01 compared with the control group; ^∗^ *p* < 0.05, ^∗∗^ *p* < 0.01, and ^∗∗∗^ *p* < 0.001 compared with the APAP group. Figure 3S: pretreatment with SAC decreased the level of CYP2E1 in APAP exposed mice. Mice were treated in APAP-induced mice. *β*-Actin served as a loading control. Mice were given PBS alone or SAC (20 mg/kg body weight) via intraperitoneal injection 1 h before challenge with APAP (400 mg/kg). Mice were killed at 12 h after APAP challenge and the liver.
