# Supplementary material for: Salvianolic Acid C against Acetaminophen-Induced Acute Liver Injury by Attenuating Inflammation, Oxidative Stress, and Apoptosis through Inhibition of the Keap1/Nrf2/HO-1 Signaling
Source: Oxid Med Cell Longev. 2019 May 12;2019:9056845. doi: 10.1155/2019/9056845 (PMC6535820; doi:10.1155/2019/9056845)
Supplement: Supplementary Materials — Figure 1S: effects of salvianolic acid C (SAC) on lipopolysaccharide- (LPS-) induced cell viability (A) and NO production (B) of RAW 264.7 macrophages. Cells were incubated for 24 h with 1 μg/mL of LPS in the absence or presence of SAC (0, 2.5, 5, 10, and 20 μg/mL). SAC was added 1 h before incubation with LPS. Cell viability assay was performed using MTT assay. Nitrite concentration in the medium was determined using Griess reagent. The data were presented as mean ± SD for the three different experiments performed in triplicate. ### compared with a sample of the control group (one-way ANOVA followed by Scheffe's multiple range tests). ∗ p < 0.05 and ∗∗∗ p < 0.001 were compared with LPS-alone group. Figure 2S: effects of SAC on GSH level in APAP-induced mice. Mice were given PBS alone or SAC (5, 10, and 20 mg/kg body weight) via intraperitoneal injection 1 h before challenge with APAP (400 mg/kg). Mice were killed at 12 h after APAP challenge and the liver. GSH was determined and expressed as μmol/g liver tissues. The values are reported as the means ± S.E.M. of five mice per group. ### p < 0.01 compared with the control group; ∗ p < 0.05, ∗∗ p < 0.01, and ∗∗∗ p < 0.001 compared with the APAP group. Figure 3S: pretreatment with SAC decreased the level of CYP2E1 in APAP exposed mice. Mice were treated in APAP-induced mice. β-Actin served as a loading control. Mice were given PBS alone or SAC (20 mg/kg body weight) via intraperitoneal injection 1 h before challenge with APAP (400 mg/kg). Mice were killed at 12 h after APAP challenge and the liver. [file 9056845.f1.docx]

1. **B.**

Figure 1S. Effects of salvianolic acid C (SAC) on lipopolysaccharide (LPS)-induced cell viability (A) and NO production (B) of RAW 264.7 macrophages. Cells were incubated for 24 h with 1 μg/mL of LPS in the absence or presence of SAC (0, 2.5, 5, 10, 20 μg/mL). SAC was added 1 h before incubation with LPS. Cell viability assay was performed using MTT assay. Nitrite concentration in the medium was determined using Griess reagent. The data were presented as mean ± SD for the three different experiments performed in triplicate. ^###^ compared with sample of control group (one-way ANOVA followed by Scheffe’s multiple range tests). **p*<0.05 and *** *p*< 0.001 were compared with LPS-alone group.

Figure 2S. Effects of SAC on GSH level in APAP-induced mice. Mice were given PBS alone or SAC (5, 10, and 20 mg/ kg body weight) via intraperitoneal injection 1 h before challenge with APAP (400 mg/kg). Mice were killed at 12 h after APAP challenge. Mice were killed at 12 h after APAP challenge and the liver. GSH was determined and expressed as μmol/g liver tissues. The values are reported as the means ± S.E.M. of five mice per group. ###p < 0.01 compared with the control group; *p < 0.05, **p < 0.01, and ***p < 0.001 compared with the APAP group.


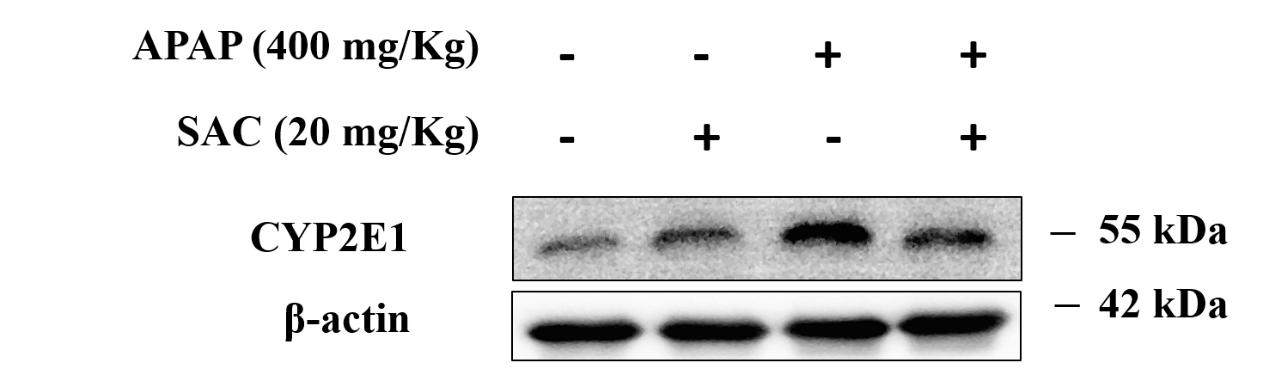


Figure 3S. Pretreatment with SAC decreased the level of CYP2E1 in APAP exposed mice. Mice were treated in APAP-induced mice. β-actin served as a loading control. Mice were given PBS alone or SAC (20 mg/ kg body weight) via intraperitoneal injection 1 h before challenge with APAP (400 mg/kg). Mice were killed at 12 h after APAP challenge. Mice were killed at 12 h after APAP challenge and the liver.
